# Military Perspectives on the Provision of Spiritual Care in the Australian Defence Force: A Cross-Sectional Study

**DOI:** 10.1007/s10943-023-01985-3

**Published:** 2024-01-22

**Authors:** Megan C. Best, Katie Tunks Leach, Mark Layson, Lindsay B. Carey

**Affiliations:** 1 Institute for Ethics and Society, University of Notre Dame Australia, PO Box 944, Broadway, NSW 2007 Australia; 2 University of Technology Sydney, Sydney, Australia; 3 St Marks National Theological Centre, Charles Sturt University, Canberra, Australia; 4 School of Psychology and Public Health, La Trobe University, Melbourne, Australia

**Keywords:** Chaplains, Military, Religion, Spirituality, Barriers

## Abstract

A module to explore perspectives on chaplaincy services was included in an online enterprise survey randomly distributed to members of the Australian Defence Force (ADF) during 2021. Up to eight questions were answered by 2783 active military personnel relating to their perception of chaplain activities and the impact of chaplaincy services. Of those military participants answering the question on religious status (*n* = 1116), a total of 71.6% (*n* = 799) of respondents identified as non-religious while 28.4% (*n* = 317) identified as holding a religious affiliation. Approximately 44.2% (*n* = 1230) of participants had sought support from a chaplain, of which 85.3% (*n* = 1049) found chaplaincy care to be satisfactory or very satisfactory. While the data suggest there is a lack of clarity around the multiple roles undertaken by chaplaincy, nevertheless respondents were just as likely to prefer chaplains for personal support (24.0%), as they were to seek help from non-chaplaincy personnel such as a non-ADF counsellor (23.2%), their workplace supervisor (23.1%) or a psychologist (21.8%). This evidence affirms that the spiritual care provided by military chaplaincy remains one of several preferred choices and thus a valued part of the holistic care provided by the ADF to support the health and wellbeing of its members.

## Introduction

The impact of military service on the emotional, psychological, social and spiritual wellbeing of military personnel can have significant adverse outcomes, including that of moral injury (MI), posttraumatic stress disorder (PTSD) and death by suicide (Jamieson et al., 2023; Koenig & Al Zaben, 2021a, ; Koenig et al., 2019; Carey & Hodgson, 2018; Ursano et al., 2015). The ripple effect of these outcomes impacts not just serving military personnel, but also their loved ones and their communities (Hodgson et al., 2021; Wright et al., 2014). One of the human resources that has consistently been available to all Australian military personnel to assist them in dealing with these challenges is that of chaplaincy. Indeed, chaplains have been involved in every military campaign of the Australian Defence Force (ADF) since its inception (Gladwin, 2013).

The association between positive spirituality/religious beliefs and wellbeing is clearly documented in the scientific literature (Garssen et al., 2016, 2021; George et al., 2011). Research has identified the benefits of maintaining spiritual and religious wellbeing to support physical, mental, and social health. These include stress reduction, significantly lower rates of depression and reduced risk of dying by suicide, lower rates of substance abuse, improved adjustment to disability, and higher rates of overall wellbeing, meaning and purpose, resilience and hope (Jones et al., 2019; Koenig, 2015; Koenig et al., 2023; Lucchese & Koenig, 2013).

Terms used to describe Australia’s spiritual and religious landscape have included ‘secular’, ‘post-secular’ and ‘pluralist’. Secularity itself is a broad movement encompassing a general decline in religious affiliation either from neutrality or opposition to religion (Taylor, 2007). Figures obtained from the Australian national census data confirm that Australians are moving away from traditional religious affiliations, with the proportion identifying as non-religious increasing from 19% in 2006 to 30% in 2016 and 38% in 2021. While Christianity still remains the most common religion in Australia (43.9%), the fastest growing religions are Hinduism (2.7%) and Islam (3.2%) (Australian Bureau of Statistics, 2022a).

In association with the changes in the religious landscape, spiritual care has been conceptualized broadly within Australian services. Spiritual Care Australia, a national professional organisation for chaplains and spiritual care practitioners in Australia, has developed standards for the delivery of spiritual care within a variety of settings (Spiritual Care Australia, 2023; Spiritual Health Association, 2020, 2022). They affirm that all people, regardless of religious faith or cultural background, have spiritual and pastoral needs that require a sensitive, respectful response from skilled practitioners and define spiritual care as encompassing:

“…. all the ways in which attention is paid to the spiritual dimension of life… is person-centred and makes no assumptions about personal conviction or life orientation. It offers a way for people to make meaning of their lived experience… Spiritual care may include presence, conversations, ritual, ceremonies and sharing of sacred texts and resources” (Spiritual Care Australia, 2023).

Extensive empirical evidence of the unique construct of spirituality, comprising features such as meaning or purpose in life, life satisfaction, inner peace, moral values, connection, and transcendent hope, underscores the requisite for trained chaplains and spiritual practitioners to meet the spiritual needs of military personnel (Armstrong, 1995; Bodling et al., 2013; Hall & Edwards, 1996; Hodge, 2003; Jackson-Lowman et al., 1996; Jagers & Smith, 1996; Koenig & Al Zaben, 2021a; Monod et al., 2011; Stewart & Koeske, 2006).

As chaplaincy has evolved over the last two decades to incorporate belief systems extending beyond the original Christian model of chaplaincy, so too has research exploring the range of activities conducted by chaplains. Programs conducted by individual chaplains and/or in collaboration with healthcare professionals such as psychologists, have been shown to better equip soldiers and promote wellbeing in advance of deployment (Koenig et al., 2022; Thomas et al., 2018), as well as support them in the theatre of war (Roberts et al., 2018), help them to recover after diagnosis of MI and/or PTSD (Ames et al., 2021; Hodgson et al., 2022; Pearce et al., 2018), and reduce veteran and service member suicide rates (Davis, 2022).

However, despite international evidence of the effectiveness of chaplaincy in the military (Layson et al., 2022), calls to review or remove chaplaincy programs from both military and public spaces more broadly, continue to be misleadingly presented in Australia and in other Western countries, with the suggestion that the declining rates of religiosity are aligned with a decreased demand and appropriateness of chaplaincy programs (Australian Bureau of Statistics, 2022a; Hoglin, 2021). A recent literature review however did not support the hypothesis that declining religiosity leads to reduced chaplaincy utilisation (Layson et al., 2022).

Most research to date has been conducted in the United States of America, and there is a lack of voices from Australian military personnel themselves expressing what they want, or what they value in their current chaplaincy support programs. The aim of this study was to investigate views of ADF personnel regarding chaplaincy services. In particular we aimed to test the following hypotheses:

1. Members of the ADF are willing to use the services offered by an ADF Chaplain
2. Members of the ADF are willing to use the services offered by an ADF Chaplain, including pastoral care, regardless of the Chaplain’s religious affiliation.
3. Members of the ADF determine whether to utilise the services offered by an ADF Chaplain using factors other than declared religion.

## Methods

### Study Design

This was a cross-sectional online survey.

### Setting

This study was conducted within a sample of the ADF across all eight Australian states and territories. The ADF comprises Navy, Army and Air Force military personnel. Eligible participants were active military personnel with current Defence email accounts and had 24-h access to full-time or reservist chaplains both at home and in war or war-like zones. The Australian Bureau of Statistics reported that at the 2021 census there were 60,286 full time military personnel and a further 24,581 part-time (military reservists) currently serving in the ADF (Australian Bureau of Statistics, 2022b). Of this cohort, 42% identified a religious affiliation and 56% did not (Roy Morgan, 2020). Approximately 300 chaplains are employed by the ADF (full time and part-time) giving an approximate ratio of 100 chaplains to every 28,289 personnel.

### Procedure

Questions were developed by the researchers MB and LC based on the academic literature to investigate military personnel’s understanding of the chaplain’s role, usage of, and satisfaction with chaplaincy services. Skip logic was used to personalise the questions asked of each participant, with a potential total of eight questions asked. As a result, not every participant was offered every question. Demographic data were collected by the parent survey and aligned with the responses to the chaplaincy specific questions (Appendix 1).

Questions were then submitted to Defence for ethical review and approval prior to inclusion in the Defence enterprise survey. In September 2021, the survey questions were sent to a selected 25% stratified random sample of the Defence workforce through an automated report system that allows selection of a cohort that is representative on the basis of Service, Organisational Group, Rank Grouping (Senior Officers, Junior Officers, Non-Commissioned Senior Officers, & Junior Non-Commissioned Officers and other ranks), State/Territory and Gender.

### Ethics Approval

This study was performed in line with the principles of the Declaration of Helsinki (1964 and subsequent amendments) and the Australian National Statement on Ethical Conduct in Human Research (NHMRC, 2018). This research was granted approval by the Departments of Defence and Veterans’ Affairs Human Research Ethics Committee (DDVAHREC 269–20).

### Data Collection

Independent of the researchers, data were collected and managed by Defence. Participants were provided a participant information form prior to commencing the study, and consent was explicitly sought for the whole survey. Privacy and confidentiality were maintained through the screening of the data by appropriate delegated Defence personnel prior to it being provided to the authors.

### Data Analysis

Survey data were received by the researchers from Defence as a Microsoft Excel.csv file and IBM SPSS Statistics.sav file. Demographic data were tabulated, and summary statistics used to describe the results. Data and findings were then analysed in line with the research hypotheses.

## Results

The survey was completed by 2,783 military participants. The cohort (Navy = 21.4%, Army = 43.3%, Air Force = 35.3%) was mostly male (80.3%). This reflects the gender distribution of the ADF. Permitted demographic details are reported in Table 1.

**Table 1:** Demographic data (*n* = 2783)

	Percentage (*n*) *
*Gender*	
Female Male Other	17.7 (401) 80.3 (1819) 2.0 (42)
Age 16–24 25–34 35–44 45–54 55–64 65 +	5.2 (111) 22.4 (480) 28.2 (606) 28.8 (617) 13.6 (291) 1.9 (41)
*Religious identity—Non-religious*	(796)
Atheist Agnostic Spiritual Not sure Prefer not to say Humanist Other	23.9 (265) 13.2 (146) 10.5 (116) 9.2 102) 8.3 (92) 3.4 (38) 3.3 (37)
*Religious identity—Religious*	(314)
Christian Buddhist Hindu Muslim Sikh Jewish Other	22.6 (251) 0.6 (7) 0.5 (6) 0.2 (2) 0.2 (2) 0.1 (1) 2.9 (32)
*State (current work location)*	
NSW Qld ACT Vic SA WA NT Overseas Tas	28.5 (635) 23.6 (526) 17.0 (379) 10.0 (223) 8.6 (191) 5.5 (123) 5.4 (120) 0.9 (20) 0.4 (10)

^*^Totals may not add up to 100% as not all participants were presented with all questions due to skip logic

**Fig. 1:**
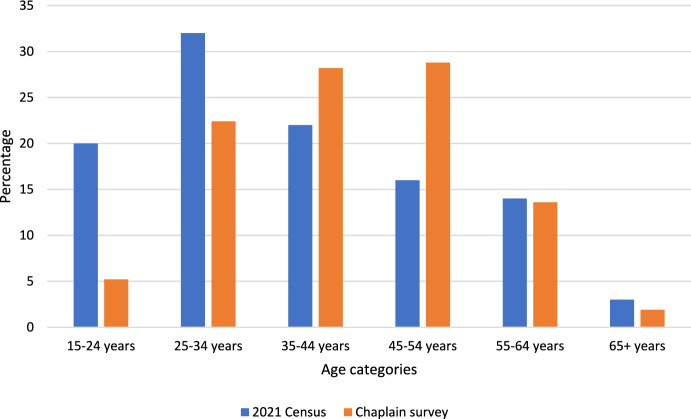
Age categories comparison between 2021 Australian Census and Chaplaincy Survey. Source for 2021 Census data (Australian Bureau of Statistics, 2022b)

The age spread of participants in the chaplaincy survey was skewed towards older age group classifications compared to ADF personnel ages reported by the ABS (Fig. 1). Therefore, data is reported for discrete age groups.

Participants were asked about their perceptions of the role of chaplains in the ADF. Most respondents were of the opinion that the main role of chaplains was to: provide general counselling and guidance (80.0%, *n* = 1859); provide support for families (79.9%, *n* = 1857); undertake religious/secular rituals (79.3%, *n* = 1843); and give advice to commanders (75.5%, *n* = 1754). Less participants were of the opinion that the chaplain’s role included: assessing members overall well-being and resilience (63.9%, *n* = 1485); conducting training\in areas such as leadership, character development and suicide assist (45.8%, *n* = 1064); or undertaking administrative and management tasks (49.1%, *n* = 1142). Lowest level of understanding about the full scope of a chaplain’s roles was in the 25–34 year age bracket (Table 2).

**Table 2:** Understanding of the role of chaplains according to age

Percentage (*n*)							
	Total	16–24	25–34	35–44	45–54	55–64	65 +
Assess wellbeing	63.9 (1485)	67.3 (70)	58.2 (269)	63.1 (377)	65.1 (398)	72.4 (210)	80.5 (33)
Counsel/guide	80.0 (1859)	86.5 (90)	81.8 (378)	80.2 (479)	78.6 (480)	79.7 (231)	90.2 (37)
Family support	79.9 (1857)	74.0 (77)	71.6 (331)	78.7 (470)	84.0 (513)	90.3 (262)	95.1 (39)
Ritual	79.3 (1843)	73.1 (76)	72.5 (335)	81.1 (484)	82.3 (503)	83.8 (243)	92.7 (38)
Advise Command	75.5 (1754)	78.8 (82)	69.3 (320)	72.5 (433)	79.7 (487)	83.8 (243)	87.8 (36)
Training	45.8 (1064)	41.3 (43)	35.1 (162)	43.2 (258)	50.1 (306)	57.9 (168)	78.0 (32)
Admin	49.1 (1142)	41.3 (43)	38.3 (177)	48.2 (288)	55.3 (338)	61.4 (178)	63.4 (26)
Other	3.9 (90)	3.8 (4)	3.8 (22)	2.7 (16)	4.3 (26)	3.1 (9)	7.3 (3)

Due to skip logic or some questions being left unanswered the sum of the age groups does not equal the total figure. The percentages reported under the age columns are the percentages for those who answered both age question and question on chaplain’s role.

When members were asked who they would prefer to see if they required personal support, responses across all age groups showed chaplains were marginally the most preferred support services; chaplains (24.0%), followed by non-ADF counsellors (23.2%), workplace supervisors (23.1%), psychologists (21.8%) and social workers (2.0%) (Table 3).

**Table 3:** Preferred support service provider according to age

	Percentage (*n*)						
	Total	16–24	25–34	35–44	45–54	55–64	65 +
Chaplain	24.0 (563)	31.5 (34)	20.0 (94)	20.6 (124)	24.5 (150)	32.9 (95)	53.7 (22)
Non ADF-counsellor	23.2 (545)	21.3 (23)	27.1 (127)	23.4 (141)	21.8 (133)	14.9 (43)	7.3 (3)
Supervisor	23.1 (542)	19.4 (21)	21.7 (102)	24.4 (147)	22.6 (138)	26.3 (76)	24.4 (10)
Psychologist	21.8 (511)	23.1 (25)	24.7 (116)	23.4 (141)	21.9 (134)	17.3 (50)	9.8 (4)
Other	6.0 (141)	4.6 (5)	5.1 (24)	6.3 (38)	6.9 (42)	5.5 (16)	2.4 (1)
Social worker	2.0 (47)	0.0 (0)	1.3 (6)	2.0 (12)	2.3 (14)	3.1 (9)	2.4 (1)

Due to skip logic or some questions being left unanswered the sum of the age groups does not equal the total. The percentages reported under the age columns are the percentages for those who answered both age question and the preferred support service

Of particular interest is that most respondents (67.8%) thought it was ‘important’ or ‘very important’ to have chaplains available in the ADF (Table 4). The age group reporting the lowest level of importance for chaplains were the 25–34 (57.3%) and 35–44 (66.0%) age groups. However, those reporting that access to chaplains was ‘unimportant’ did not rise above 17.5% (25–34 age group).

**Table 4:** Reported importance of chaplaincy according to age

	Percentage (*n*)						
	Total	16–24	25–34	35–44	45–54	55–64	65 +
Very important	34.9 (823)	35.8 (39)	26.3 (125)	29.7 (180)	39.9 (245)	49.0 (143)	75.0 (30)
Important	32.9 (776)	35.8 (39)	30.9 (147)	36.3 (220)	32.6 (200)	32.2 (94)	12.5 (5)
Not sure/Indifferent	19.6 (463)	20.2 (22)	25.3 (120)	17.7 (107)	17.6 (108)	13.4 (39)	10.0 (4)
Not important	6.9 (163)	4.6 (5)	7.6 (36)	10.2 (62)	5.4 (33)	2.4 (7)	2.5 (1)
Not at all important	5.7 (135)	3.7 (4)	9.9 (47)	6.1 (37)	4.6 (28)	3.1 (9)	0.0 (0)

Due to skip logic or some questions being left unanswered the sum of the age groups does not equal the total. The percentages reported under the age columns are the percentages for those who answered both age question and the importance of chaplaincy

Over 40% of the cohort (44.2%, *n* = 1230) had previously sought assistance from an ADF chaplain. When they were asked about their satisfaction with the support they received, the majority (85.3%, *n* = 1049) reported they were either satisfied or very satisfied with the support they received (Table 5). Levels of satisfaction according to age ranged from 75.6% (16–24 age group) to 100% (65 + age group).

**Table 5:** Satisfaction with support provided by chaplains

Percentage (*n*)							
	Total	16–24	25–34	35–44	45–54	55–64	65 +
Very satisfied	43.0 (529)	39.0 (16)	33.3 (72)	35.2 (120)	50.9 (179)	55.6 (89)	78.6 (22)
Satisfied	42.3 (520)	36.6 (15)	48.6 (105)	48.1 (164)	39.2 (138)	35.6 (57)	21.4 (6)
Neither	9.7 (119)	14.6 (6)	12.0 (26)	11.4 (39)	6.5 (23)	7.5 (12)	0.0 (0)
Unsatisfied	2.3 (28)	4.9 (2)	1.9 (4)	2.9 (10)	1.7 (6)	0.6 (1)	0.0 (0)
Very unsatisfied	2.8 (34)	4.9 (2)	4.2 (9)	2.3 (8)	1.7 (6)	0.6 (1)	0.0 (0)

Due to skip logic or some questions being left unanswered the sum of the age groups does not equal the total. The percentages reported under the age columns are the percentages for those who answered both age question and the satisfaction level question

Those that had received support were asked why they had sought chaplaincy support. The top six reasons for selecting chaplains were because: (1) chaplains were able to discuss personal/family issues (42.6%, *n* = 522); (2) chaplains are part of the ADF and understand pressures on military members and families (42.2%, *n* = 516); (3) chaplains are able to discuss/understand ADF workplace issues (29.2%, *n* = 357); (4) chaplains are accessible at all times (27.9%, *n* = 342); (5) chaplains have been helpful in the past (27.9%, *n* = 341); and (6) chaplains are able to assist with rituals such as weddings, funerals, etcétera. (24.3%, n = 297).

Regarding other reasons for seeking chaplain support, there  was a downward linear trend between age and likelihood of accessing chaplains based on a recommendation with 30.8% of the 16–24 age group seeking support on recommendation down to 3.6% for those aged over 65. Conversely, those who had previously found chaplaincy helpful increased with age from 20.5% of 16–25 respondents up to 53.6% in those over 65. So too, the utilization of chaplains for ceremonies increased in a linear fashion with age from 5.1% in the 16–24 age group, up to 41.0% in the 54–65 age group. Those who sought chaplains for spiritual guidance was highest in the 45–54 age group (29.3%) and the 16–24 age group (17.9%). There was a pronounced dip in the utilization of chaplains for spiritual guidance in the 25–34 age group (10.7%) and 35–44 age group (10.6%) (Table 6).

**Table 6:** Reasons for seeking support from chaplains

Percentage (*n*)							
	Total	16–24	25–34	35–44	45–54	55–64	65 +
Always accessible	27.9 (342)	35.9 (14)	26.6 (57)	25.7 (87)	29.1 (102)	29.2 (47)	42.9 (12)
Recommended	13.9 (170)	30.8 (12)	23.4 (50)	13.6 (46)	9.4 (33)	8.1 (13)	3.6 (1)
Family issues	42.6 (522)	41.0 (16)	39.3 (84)	41.3 (140)	45.0 (158)	44.1 (71)	64.3 (18)
Financial issues	4.2 (51)	2.6 (1)	2.8 (6)	4.1 (14)	4.6 (16)	6.2 (10)	0.0 (0)
Workplace issues	29.2 (357)	23.1 (9)	25.7 (55)	30.7 (104)	29.3 (103)	29.8 (48)	28.6 (8)
Spiritual guidance	13.3 (163)	17.9 (7)	10.7 (23)	10.6 (36)	29.3 (103)	13.0 (21)	25.0 (7)
Ceremonies	24.3 (297)	5.1 (2)	13.1 (28)	19.8 (67)	29.9 (105)	41.0 (66)	39.3 (11)
Understand military service	42.2 (516)	33.3 (13)	36.9 (79)	39.5 (134)	43.6 (153)	48.4 (78)	67.9 (19)
Helpful in past	27.9 (341)	20.5 (8)	23.8 (51)	25.7 (87)	31.1 (109)	31.7 (51)	53.6 (15)
Other	13.2 (161)	7.7 (3)	14.0 (30)	13.3 (45)	14.0 (49)	9.9 (16)	14.3 (4)

Due to skip logic or some questions being left unanswered the sum of the age groups does not equal the total. The percentages reported under the age columns are the percentages for those who had sought support, answered the age question and the reasons for seeking support

**Fig. 2:**
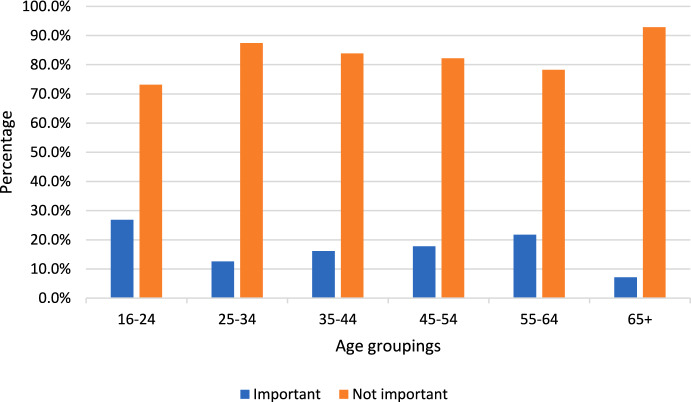
Importance of the religion of the chaplain

Those who had previously accessed military chaplains were asked if the religion of the chaplain was important to them and 82.8% (*n* = 1020) reported that it was ‘not important’. Figure 2 displays how this varied across age groups.

Those who had not previously sought support from military chaplains were asked why this was the case. The reasons for this are reported in Table 7. Respondents reported not needing to discuss personal/family issues (59.2%, *n* = 651), not needing spiritual guidance or counsel (50.3%, *n* = 553), and not needing to discuss workplace issues (48.7%, *n* = 535). Less prevalent reasons included not wanting to talk to a religious person (25.5%, *n* = 280), personnel preferring to solve issues by themselves (19.7%, *n* = 213), preferring to access help from outside the ADF (19.4%, *n* = 213), and preferring to speak to a secular counsellor (13.6%, *n* = 150). There was a linear association between age and preferring help outside of the ADF, with 31.8% of the 16–24 age group preferring help from outside the ADF to 0% for those aged over 65. Those in the 35–44 and 45–54 age groups were the most likely to not want to speak to a religious person and seek support from a secular counsellor (Table 7).

**Table 7:** Reasons for not seeking chaplain support

Percentage (*n*)							
	Total	16–24	25–34	35–44	45–54	55–64	65 +
Not available	2.7 (30)	3.0 (2)	3.5 (9)	2.3 (6)	3.1 (8)	3.1 (4)	0.0 (0)
No recommendation	2.6 (29)	7.6 (5)	2.8 (7)	1.9 (5)	1.6 (4)	1.6 (2)	7.7 (1)
Uncertain of role	2.4 (26)	3.0 (2)	2.8 (7)	3.1 (8)	0.8 (2)	0.0 (0)	0.0 (0)
Not needed for family issues	59.2 (651)	72.7 (48)	55.1 (140)	54.2 (141)	64.6 (166)	64.8 (83)	76.9 (10)
Not needed for financial issues	40.2 (442)	57.6 (38)	39.4 (100)	33.5 (87)	43.2 (111)	43.0 (55)	53.8 (7)
Not needed for workplace issues	48.7 (535)	60.6 (40)	50.4 (128)	43.8 (114)	49.0 (126)	53.1 (68)	53.8 (7)
Not needed for spiritual guidance	50.3 (553)	60.6 (40)	50.8 (129)	48.5 (126)	52.1 (134)	49.2 (63)	61.5 (8)
Not needed for ceremony	39.6 (435)	56.1 (37)	39.4 (100)	33.5 (87)	40.9 (105)	43.8 (56)	53.8 (7)
Concerned about confidentiality	8.7 (96)	15.2 (10)	12.6 (32)	7.7 (20)	5.8 (15)	6.3 (8)	7.7 (1)
Don’t want a religious person	25.5 (280)	19.7 (13)	24.0 (61)	29.32(76)	31.1 (80)	13.3 (17)	15.4 (2)
Rather speak to secular counsellor	13.6 (150)	13.6 (9)	11.8 (30)	15.4 (40)	15.6 (40)	7.8 (10)	7.7 (1)
Prefer solve issues myself	19.7 (121)	19.7 (13)	11.8 (30)	8.8 (23)	10.1 (26)	14.1 (18)	7.7 (1)
Prefer help from outside ADF	19.4 (213)	31.8 (21)	25.2 (64)	18.5 (48)	14.0 (36)	11.7 (15)	0.0 (0)
Not helpful in past	7.9 (87)	7.6 (5)	9.8 (25)	9.2 (24)	7.0 (18)	2.3 (3)	0.0 (0)
Other	3.8 (42)	3.0 (2)	3.5 (9)	3.1 (8)	5.4 (14)	2.3 (3)	0.0 (0)

Due to skip logic or some questions being left unanswered, the sum of the age groups does not equal the total. The percentages reported under the age columns are the percentages for those who had not sought support, answered the age question, and the reasons for not seeking support

## Discussion

The aim of this study was to investigate ADF military perspectives regarding military chaplaincy services to better understand what they perceive the chaplaincy role to be, the value they place on chaplaincy care, and satisfaction with this care. Furthermore, it sought to understand whether barriers to accessing chaplaincy care exist by asking personnel who had not used chaplaincy, why this was the case.

### Hypotheses

This study sought to test three hypotheses. The first was whether military personnel of the ADF valued the services offered by an ADF Chaplain. The simple answer is ‘Yes’. Over 40% of the respondents in this survey (44.2%) had previously accessed chaplaincy support and 67.8% believed chaplaincy was important or very important for the ADF. This data supports the first hypothesis.

Given that approxiamtely 68% believed chaplaincy was important for the ADF this random sample suggests that religiosity, or non-religiosity, of military personnel may not be a pre-determinant for valuing or accessing military chaplains, which is consistent with the findings of previous literature (Layson et al., 2022). This may reflect the universality of spirituality and spiritual needs, which may be overlooked by those who confuse spirituality with religion, and/or may be unaware of the evidence for the unique nature of the construct of spirituality and spiritual need. It is also known that, despite identifying as ‘non-religious’, this subset of the population is known to have a dynamic relationship with religion which may be more prominent in times of duress (Davie, 2015; Van Tongeren et al., 2021). Increased interest in chaplaincy support at times of struggle has been demonstrated in the healthcare setting (Best et al., 2023). This should be further explored in the military context.

However, it is important to qualify that the majority of respondents who had not previously engaged with a chaplain self-identified as ‘not religious’. Respondents identifying as ‘agnostic’ or ‘spiritual’ made up 32.9% of the ‘non-religious’ respondents. Of these, 14.6% of respondents in this survey identified as ‘spiritual’ which was consistent with a previous study of Australian adults (McCrindle & Spiritual Health Association, 2021) whose findings suggest the traditional ‘religious or not religious’ dichotomy may no longer be fit for purpose as Gen Y and Gen Z embrace spirituality at rates greater than those in the Gen X and Baby Boomers (McCrindle & Spiritual Health Association, 2021). How these findings correlate to religion is as yet unknown, so basing future predictions on religious trends may not be appropriate moving forward.

Our second hypothesis was whether military personnel are willing to use chaplaincy services regardless of the Chaplain’s professional religious affiliation. We found that, of those who had accessed chaplain support, 82.8% reported that the religion of the chaplain was not important, once again supporting this hypothesis and in accordance with the international literature (Layson et al., 2022). In considering the 17.2% of respondents who did prefer a chaplain of a particular faith, this finding is not surprising, given that religion is a recognised source of spiritual support for a proportion of the Defence population, and, as previously noted, 42% of personnel at the time of the survey identified a religious affiliation (Koenig, 2015). Specifically religious needs, such as those involving ritual, may be met only by chaplains of concurrent faith, which is why faith-based chaplaincy will be an ongoing requirement to meet the needs of some personnel. Jansson has noted that secular humanism, regardless of the form in which it is presented, is insufficient to address all  forms of spiritual need (Jansson, 1990).

Our third hypothesis considered whether members of the ADF had determined to utilize the services offered by an ADF Chaplain using factors other than declared religion. The role of faith concordance is important to address in a pluralistic society like Australia. We found that the primary motivation for accessing ADF chaplains related to their accessibility and their ability to relate to the issues directly impacting on ADF personnel. Similarly, those who had not accessed chaplains (similar to the findings of hypothesis 2) also listed religiosity as a minor consideration. This is consistent with literature in other disciplines. In the healthcare context, some authors have described religious and cultural diversity as a challenge, with potential risks including staff (irrespective of being religious or non-religious) inadvertently imposing their beliefs on clients or offending them when conflicts in belief systems emerge (Best et al., 2016; Hodge & Lietz, 2014). However, a Dutch study found that patients in faith-concordant encounters and faith-discordant encounters evaluated spiritual care encounters with equal positivity (Liefbroer & Nagel, 2021). This would reflect the growing professionalisation of chaplaincy enabling them as spiritual care practitioners to engage with all patients regardless of faith (Pesut et al., 2016; Stifoss-Hanssen et al., 2019).

Another noteworthy finding in this study was that respondents valued chaplaincy similarly to other support options including psychology, non-ADF counsellors and supervisors. Despite the negative critique of some commentators strongly opposed to chaplaincy, ADF military personnel in this study suggested chaplaincy was equally valued alongside current  options, and in fact chaplaincy was significantly more selected than social workers (24% versus 2%). This latter finding may reflect the reduced access to social workers across the ADF. However, our data also indicates the fact that military staff have a variety of personal needs which require a diverse range of preferred support options.

Healthcare research demonstrates that spiritual wellbeing is a unique construct, independent of social, psychological, and physical factors and positively associated with quality of life (Brady et al., 1999). As such, spiritual care will be at times a unique, distinct, and definite need for military personnel, that cannot usually be met by other professions. In order to respect the autonomy of personnel, it is necessary for the ADF to maintain a range of choices, thereby empowering staff to make decisions about their own wellbeing. This appears to be a novel finding and requires further investigation.

Of those who had received chaplaincy care, 85.3% rated chaplaincy care as ‘satisfactory’ or ‘very satisfactory’ with only 5.0% rating it as unsatisfactory. Data evaluating other staff support services in the ADF are scarce, making it difficult to determine if this finding is comparable to offerings including psychology and non-ADF counsellors. While this figure suggests there is some room for improvement, chaplaincy appears to be meeting the needs of most of those who choose to use it as a support service.

We found that chaplaincy was least important among the 25–34 years age group (57.3%), a sub-cohort which also had the lowest level of awareness regarding the role of chaplains. The literature on chaplaincy also reports that a lack of understanding about the role of chaplains is a noted barrier to chaplaincy utilization (Layson et al., 2022). It is possible therefore that with increased understanding about chaplaincy competency and capability, the proportion of personnel accessing chaplaincy services could increase.

We also found that the reasons given for personnel not previously seeking chaplaincy support related to personnel not needing any support, or a desire to seek support entirely outside the ADF. It is reasonable to expect that not all personnel will require support from a chaplain, hence the need for a range of support options in the workplace which is currently the ADF practice. While some personnel reported not wanting to talk to a religious person, it is well known that confusion between religion and spirituality and a lack of understanding about the broad role of chaplains, may act as a barrier to accessing spiritual care in Western communities when it is associated with previous negative experiences with religion (Best et al., 2023; Layson et al., 2022). These findings suggest that increased education regarding the holistic biopsychosocial-spiritual nature of pastoral care would be of benefit to military personnel.

### Limitations

This is a cross-sectional survey which reflects the opinions of the cohort at one point in time. Qualitative research in the future would allow deeper understanding of the reasons for the views presented. There was also a disparity in ages between those who answered the survey and the ADF population. This limitation was somewhat overcome by analysing data from each age group in detail, however, it would be helpful in future research if a much larger sample of ADF military personnel were recruited which would add to the quality of the findings and enable generational trends to be analysed in relation to chaplaincy utilization. It would also be advantageous if future research was able to discern any differences between part-time and full-time military personnel, as well as between Army, Navy and Air Force personnel, as there could be differences in ease of access to chaplains. Future research could also consider the role of the Religious Advisory Committee to the Services (RACS) regarding chaplain recruitment, although current research indicates that faith-based chaplaincy within the ADF is fit for purpose (Layson et al., 2023). Despite these limitations, the survey results provide valuable and unique insight into the views of ADF military personnel.

## Conclusions

This study confirmed our hypotheses that ADF personnel used military chaplaincy regardless of the chaplain’s religious beliefs, and for factors other than religion. Most participants in this study believed chaplaincy was an important element of ADF support and were satisfied with the support they received. Our findings clearly challenge the idea that faith similarity between chaplain and military personnel is necessary to provide this care—in fact quite the opposite—and that military personnel are aware that the support provided by chaplains extends beyond religious boundaries.

Challenges, however, for ADF chaplaincy remain, including the lack of clarity surrounding the chaplain’s role and activities. Nevertheless, while no single staff support option can meet the needs of all members, findings from this study suggest that for those who use military chaplaincy, it remains a valued and effectively high satisfaction option for meeting personal and professional needs.

## Appendix 1: Survey Questions

1. What do you think is the role of a Defence Chaplain? (Please tick all options that apply)
  o Assessing defence members overall wellbeing and resilience given circumstances and resources
  o General counselling, guidance, and education to assist members and their family to cope and make decisions given complex situations (involving topics such as workplace issues, relationship issues, ethical issues)
  o Support to families by being present or advocating for members and their family – particularly at times of workplace issues, loss of meaning, injury, death, bereavement ad grieving.
  o Rituals (e.g., prayer, weddings, funerals, ceremonial/memorial occasions like ANZAC Day, multifaith religious services).
  o Training (e.g., leadership and character development programs, suicide assist).
  o Advice (e.g., commander advice on moral, ethical and personnel matters; advises on host nation and enemy combatant religious, spiritual, ethical issues as required, undertakes Religious Leader Engagement with host nations normally during peace support operations or in post-conflict environments).
  o Administration and Management (e.g., liaising with local community; undertakes Religious Leader Engagement with host nations, organising of support for personnel irrespective of rank or occupation.)
  o Other (please give details)_______________________________________
2. If you needed personal support from within defence, who would you prefer to see?
  o Chaplain
  o Psychologist
  o Social worker
  o Supervisor
  o Non-ADF counsellor
  o Other (please specify) _______________________________________
  o Comments (optional)________________________________________
3. How important do you think it is to have chaplains available in the ADF?

**Table:**

Very important	Important	Not sure/indifferent	Not important	Not at all important

4. Have you ever sought help/support from, or been given help/support by, a Defence Chaplain?
  o Yes
  o No

## Two Streams of Questions, Depending on Answer to Question 4:

### Stream 1) YES, I Have Previously been Supported by a Defence Chaplain

5. Why did you seek support from a chaplain? Mark all that apply:
  - They are accessible at all times
  - Someone recommended the chaplain
  - Needed someone to confidentially discuss personal/family issues
  - Needed some to confidentially discuss financial issues
  - Needed someone to confidentially discuss workplace issues
  - Needed spiritual guidance or advice
  - I needed assistance with funeral, wedding, baptism, or memorial service
  - Chaplains are part of the ADF and understand both civilian and military pressures on service personnel and their families.
  - I found them helpful in the past
  - Other—please specify
  6. Was the particular religion of the chaplain important to you? Yes/No
  - Comments (optional)_____________________________________________
  7. When you have met with a chaplain, were you satisfied with their help/support? (Please mark one)

**Table:**

Very satisfied	Satisfied	Neither satisfied nor unsatisfied	Unsatisfied	Very unsatisfied

- Comments (optional)__________________________

### Stream 2) NO, I have Not Previously been Supported by a Defence Chaplain

5. Why have you NOT sought the support or assistance of a chaplain? (Please tick all that apply)

- There have been no chaplains available
- No one has ever recommended the chaplain
- I’m uncertain about what chaplains do
- I have not needed a chaplain to discuss personal/family issues
- I have not needed a chaplain to discuss financial issues
- I have not needed a chaplain to discuss workplace issues
- I have not needed spiritual guidance or counsel
- I have not needed assistance with a funeral, wedding, or memorial service.
- I am concerned about trust and confidentiality
- I don’t want to talk to a religious person
- I would rather talk to a secular counsellor
- I like to do things without help from anybody
- I would rather seek help outside the defence force
- I have not found them helpful in the past
- Other (please specify)____________________________________
- Comments (optional)____________________________________

8/6* Do you have a current religious affiliation?

- Yes
- No

If yes, please select from the following:

o Buddhist
o Christian
o Hindu
o Jewish
o Muslim
o Sikh
o Prefer not to say
o Not sure
o Other (please specify)

If no, which might describe you best from the following:

o Agnostic
o Atheist
o Humanist
o Spiritual but not religious
o Not sure
o Prefer not to say
o Other (please specify)

*Question 8/6: The survey was designed to merge into one stream at this point. Due to an error in data collection, this question was not presented to participants who had previously been supported by a Defence chaplain.

## References

[CR1] Ames D, Erickson Z, Geise C, Tiwari S, Sakhno S, Sones AC, Tyrrell CG, Mackay CRB, Steele CW, Van Hoof T, Weinreich H, Koenig HG (2021). Treatment of moral injury in U.S. veterans with PTSD using a structured chaplain intervention. Journal of Religion and Health.

[CR2] Armstrong, T. D. (1995). Exploring spirituality: The development of the Armstrong measure of spirituality. Annual convention of the American psychological association, New York, NY.

[CR3] Australian Bureau of Statistics. (2022b). *Australian Defence Force service*. Australian Bureau of Statistics. Retrieved Aug 13 2023 from https://www.abs.gov.au/articles/australian-defence-force-service#:~:text=Currently%20serving%20ADF%20members%20have,aged%2065%20years%20or%20more.

[CR4] Australian Bureau of Statistics. (2022a). *2021 Census shows changes in Australia’s religious diversity*. https://www.abs.gov.au/media-centre/media-releases/2021-census-shows-changes-australias-religious-diversity.

[CR5] Best MC, Butow P, Olver I (2016). Creating a safe space: A qualitative inquiry into the way doctors discuss spirituality. Palliative and Supportive Care.

[CR6] Best MC, Jones K, Merritt F, Casey M, Lynch S, Eisman JA, Cohen J, Mackie D, Beilharz K, Kearney M (2023). Australian patient preferences for discussing spiritual issues in the hospital setting: An exploratory mixed methods study. Journal of Religion and Health.

[CR7] Bodling A, Heneghan M, Walsh JC, Yoon DP, Johnstone B (2013). The brief multidimensional measure of religiousness/spirituality with an irish sample: A factor analysis. International Journal of Therapy and Rehabilitation.

[CR102] Brady, M. J., Peterman, A. H., Fitchett, G., Mo, M., & Cella, D. (1999). A case for including spirituality in quality of life measurement in oncology. *Psycho-Oncology, 8*(5), 417–428. 10.1002/(SICI)1099-1611(199909/10)8:5<417::AID-PON398>3.0.CO;2-4 10.1002/(sici)1099-1611(199909/10)8:5<417::aid-pon398>3.0.co;2-4 10559801

[CR506] Carey, L. B., & Hodgson, T. J. (2018). Chaplaincy, spiritual care and moral injury: Considerations regarding screening and treatment. *Frontiers in Psychiatry, 9*(619), 1–10. 10.3389/fpsyt.2018.00619 10.3389/fpsyt.2018.00619 PMC6290645 30568605

[CR8] Garssen B, Visser A, de Jager Meezenbroek E (2016). Examining whether spirituality predicts subjective well-being: How to avoid tautology. Psychology of Religion and Spirituality.

[CR9] Garssen B, Visser A, Pool G (2021). Does spirituality or religion positively affect mental health? Meta-analysis of longitudinal studies. The International Journal for the Psychology of Religion.

[CR10] George LK, Larson DB, Koenig HG, McCullough ME (2011). Spirituality and health: What we know, what we need to know. Journal of Social and Clinical Psychology.

[CR11] Gladwin M (2013). Captains of the soul: A history of Australian army chaplains.

[CR12] Hall TW, Edwards KJ (1996). The initial development and factor analysis of the spiritual assessment inventory. Journal of Psychology and Theology.

[CR13] Hodge DR (2003). The intrinsic spirituality scale. Journal of Social Service Research.

[CR14] Hodge DR, Lietz CA (2014). Using spiritually modified cognitive-behavioral therapy in substance dependence treatment: Therapists’ and clients’ perceptions of the presumed benefits and limitations. Health and Social Work.

[CR15] Hodgson TJ, Carey LB, Koenig HG (2021). Moral injury, Australian veterans and the role of chaplains: An exploratory qualitative study. Journal of Religion & Health.

[CR16] Hodgson TJ, Carey LB, Koenig HG (2022). Moral injury, betrayal and retribution: Australian veterans and the role of chaplains. Journal of Religion and Health.

[CR17] Hoglin P (2021). Secularism and pastoral care in the Australian Defence Force. Australian Army Journal.

[CR18] Jackson-Lowman H, Rogers JA, Zhang X, Zhao Y, Brathwaite-Tull M (1996). Life attitude inventory: preliminary evaluation of a measure of spiritual orientation. Handbook of tests and measurements for black populations.

[CR19] Jagers RJ, Smith P (1996). Further examination of the spirituality scale. Journal of Black Psychology.

[CR505] Jamieson, N., Carey, L. B., Jamieson, A., & Maple, M. (2023). Examining the association between moral injury and suicidal behavior in military populations: A systematic review. *Journal of Religion and Health, 62*, 3904–3925. 10.1007/s10943-023-01885-6 10.1007/s10943-023-01885-6 37592186

[CR20] Jansson J (1990). Diskussionsinlägg inom forskarutbildningen i vårdvetenskap vid Institutionen för vårdvetenskap.

[CR21] Jones KF, Simpson G, Briggs L, Dorsett P, Anderson M (2019). A study of whether individual and dyadic relations between spirituality and resilience contribute to psychological adjustment among individuals with spinal cord injuries and their family members. Clinical Rehabilitation.

[CR22] Koenig, H. G. (2015). Religion, spirituality, and health: A review and update. *Advances in Mind-Body Medicine,**29*(3), 19–26. https://europepmc.org/article/med/26026153 26026153

[CR23] Koenig H, Al Zaben F (2021). Moral injury: An increasingly recognized and widespread syndrome. Journal of Religion and Health.

[CR25] Koenig H, Carey LB, Al Zaben F (2022). Spiritual readiness: Essentials for military leaders and chaplains.

[CR26] Koenig HG, VanderWeele T, Peteet JR (2023). Handbook of religion and health.

[CR27] Koenig H, Youssef N, Ames D, Oliver R, Volk F, Teng E, Hill T (2019). Dimensions of religiosity and PTSD symptom clusters in US veterans and active duty military. Journal of Religion and Health.

[CR28] Layson, M., Carey, L.B, & Best, M. (2023). Now more than ever:“Fit for purpose”. *Australian Army Chaplaincy Journal 2023,* p. 11–12. https://nla.gov.au/nla.obj-3251324262/view.

[CR29] Layson MD, Tunks Leach K, Carey LB, Best MC (2022). Factors influencing military personnel utilizing chaplains: A literature scoping review. Journal of Religion and Health.

[CR30] Liefbroer AI, Nagel I (2021). Does faith concordance matter? A comparison of clients’ perceptions in same versus interfaith spiritual care encounters with chaplains in hospitals. Pastoral Psychology.

[CR31] Lucchese F, Koenig H (2013). Religion, spirituality and cardiovascular disease: Research, clinical implications, and opportunities in Brazil. Brazilian Journal of Cardiovascular Surgery.

[CR32] McCrindle, & Spiritual Health Association. (2021). *The future of spiritual care in Australia: A national study on spirituality, wellbeing and spiritual care in hospitals*. https://www.spiritualhealth.org.au/reports

[CR33] Monod S, Brennan M, Rochat E, Martin E, Rochat S, Büla CJ (2011). Instruments measuring spirituality in clinical research: A systematic review. Journal of General Internal Medicine.

[CR34] Pearce M, Haynes K, Rivera NR, Koenig HG (2018). Spiritually integrated cognitive processing therapy: A new treatment for post-traumatic stress disorder that targets moral injury. Global Advances in Health and Medicine.

[CR35] Pesut B, Sinclair S, Fitchett G, Greig M, Koss SE (2016). Health care chaplaincy: A scoping review of the evidence 2009–2014. Journal of Health Care Chaplaincy.

[CR36] Roberts DL, Kovacich J, Rivers MJ (2018). The comprehensive female soldier support model. Journal of Health Care Chaplaincy.

[CR100] Roy Morgan. (2020). Defence Census 2019: Public Report. Commonwealth of Australia. https://www.defence.gov.au/about/accessing-information/defence-census

[CR37] Spiritual Health Association. (2020). *Capability framework for spiritual care practitioners in health*. https://www.spiritualhealth.org.au/download/Capability_Framework_Nov2021.pdf?downloadable=1.

[CR38] Spiritual Health Association. (2022). *Standards*. Spiritual Health Association. Retrieved 20 Apr 2023 from https://www.spiritualhealth.org.au/standards.

[CR39] Spiritual care Australia (2023). Capabilities framework for spiritual care practitioners.

[CR40] Stewart C, Koeske GF (2006). Research: A preliminary construct validation of the multidimensional measurement of religiousness/spirituality instrument: A study of southern USA samples. The International Journal for the Psychology of Religion.

[CR41] Stifoss-Hanssen H, Danbolt LJ, Frøkedal H (2019). Chaplaincy in northern Europe: An overview from Norway. Tidsskrift for Praktisk Teologi.

[CR42] Taylor C (2007). A secular age.

[CR43] Thomas KH, McDaniel JT, Albright DL, Fletcher KL, Koenig HG (2018). Spiritual fitness for military veterans: A curriculum review and impact evaluation using the duke religion index (DUREL). Journal of Religion and Health.

[CR44] Ursano RJ, Kessler RC, Stein MB, Naifeh JA, Aliaga PA, Fullerton CS, Sampson NA, Kao T-C, Colpe LJ, Schoenbaum M, Cox KL, Heeringa SG (2015). Suicide attempts in the US army during the wars in Afghanistan and Iraq, 2004 to 2009. JAMA Psychiatry.

[CR45] Wright KM, Foran HM, Wood MD (2014). Community needs among service members after return from combat deployment. Journal of Community Psychology.

[CR101] Van Tongeren, D. R., DeWall, C. N., Chen, Z., Sibley, C. G., & Bulbulia, J. (2021). Religious residue: Cross-cultural evidence that religious psychology and behavior persist following deidentification. *Journal of Personality and Social Psychology, 120*(2), 484–503. 10.1037/pspp0000288 10.1037/pspp0000288 32162932

